# Netrin-1 – DCC Signaling Systems and Age-Related Macular Degeneration

**DOI:** 10.1371/journal.pone.0125548

**Published:** 2015-05-07

**Authors:** John Paul SanGiovanni, Jing Chen, Ankur S. Gupta, Lois E. H. Smith, Przemyslaw Sapieha, Phil H. Lee

**Affiliations:** 1 National Institute of Alcohol Abuse and Alcoholism, Section on Nutritional Neuroscience, National Institutes of Health, Bethesda, MD, United States of America; 2 Department of Ophthalmology, Harvard Medical School, Boston Children’s Hospital, Boston, MA, United States of America; 3 University of Texas Southwestern Medical Center at Dallas, Dallas, Texas, United States of America; 4 Department of Ophthalmology, Maisonneuve-Rosemont Hospital Research Centre, University of Montreal, Montreal, QC, Canada; 5 Analytic & Translational Genetics Unit, Center for Human Genetic Research, Massachusetts General Hospital, Harvard Medical School, Boston, MA, United States of America; Bascom Palmer Eye Institute, University of Miami School of Medicine;, UNITED STATES

## Abstract

We conducted a nested candidate gene study and pathway-based enrichment analysis on data from a multi-national 77,000-person project on the molecular genetics of age-related macular degeneration (AMD) to identify AMD-associated DNA-sequence variants in genes encoding constituents of a netrin-1 (NTN1)-based signaling pathway that converges on DNA-binding transcription complexes through a 3'-5'-cyclic adenosine monophosphate-calcineurin (cAMP-CN)-dependent axis. AMD-associated single nucleotide polymorphisms (SNPs) existed in 9 linkage disequilibrium-independent genomic regions; these included loci overlapping *NTN1* (rs9899630, *P* ≤ 9.48 x 10^-5^), *DCC* (Deleted in Colorectal Cancer)—the gene encoding a primary NTN1 receptor (rs8097127, *P* ≤ 3.03 x 10^-5^), and 6 other netrin-related genes. Analysis of the NTN1-DCC pathway with exact methods demonstrated robust enrichment with AMD-associated SNPs (corrected *P*-value = 0.038), supporting the idea that processes driven by NTN1-DCC signaling systems operate in advanced AMD. The NTN1-DCC pathway contains targets of FDA-approved drugs and may offer promise for guiding applied clinical research on preventive and therapeutic interventions for AMD.

## Introduction

Age-related macular degeneration (AMD) is a complex[1] and common[2] sight-threatening condition affecting the neural and vascular retina.[3] Park *et al*. have discussed the role of netrin-1 as a common cue for axonal guidance and vascular patterning[4], through enhancement of endothelial responses to vascular endothelial growth factor (VEGF).[5] Netrin-1 is an evolutionarily conserved signaling protein with the capacity to activate systems responsible for integrating regulatory cues from retinal neurons, glia, immune cells and vessels affected in AMD.[6, 7] Cell signaling systems activated by netrin-1 influence processes implicated in AMD pathogenesis;[3] these include cell migration, cell-substrate adhesion, elaboration of vascular networks, inflammation, and protein translation.[7]

People with AMD may have dysregulated immune and inflammatory responses, as well as altered retinal energy metabolism.[3] In retinopathy, metabolic compromise limits neuronal production of netrin-1 and prevents adequate physiological revascularization driven by microglia.[6] This netrin-1 deficiency results in shunting of metabolic resources to optimize cell survival in viable retinal cells.[6, 8] In 2008 Edwards *et al*. reported existence of AMD-associated single nucleotide polymorphisms (SNPs) (*P* < 10^–4^) in the gene encoding Deleted in Colorectal Cancer (*DCC*), the primary netrin-1 receptor.[9] A sequence variant in this same gene (rs9966348) was recently identified as one of the 20 most highly interconnected AMD-associated SNPS in a genome-wide Boolean interaction analysis involving 137.5 million tests on epistatic (SNP-SNP) relationships.[10] A clearer understanding of the capacity for netrin-1-DCC-based systems to operate efficiently on neural-, vascular-, and immune cell-driven systems in ambient[11] microenvironments may be germane for the study of AMD, as the natural history of this disease is characterized often by a circumscribed sparing of areas in the central retina until the most advanced stages of degeneration—this pattern of AMD progression has been attributed to an optimal channeling of resources to support a high metabolic load necessary to preserve homeostasis in the fovea, a retinal region involved in visual sensing of the finest features of our environment.

**Fig 1:**
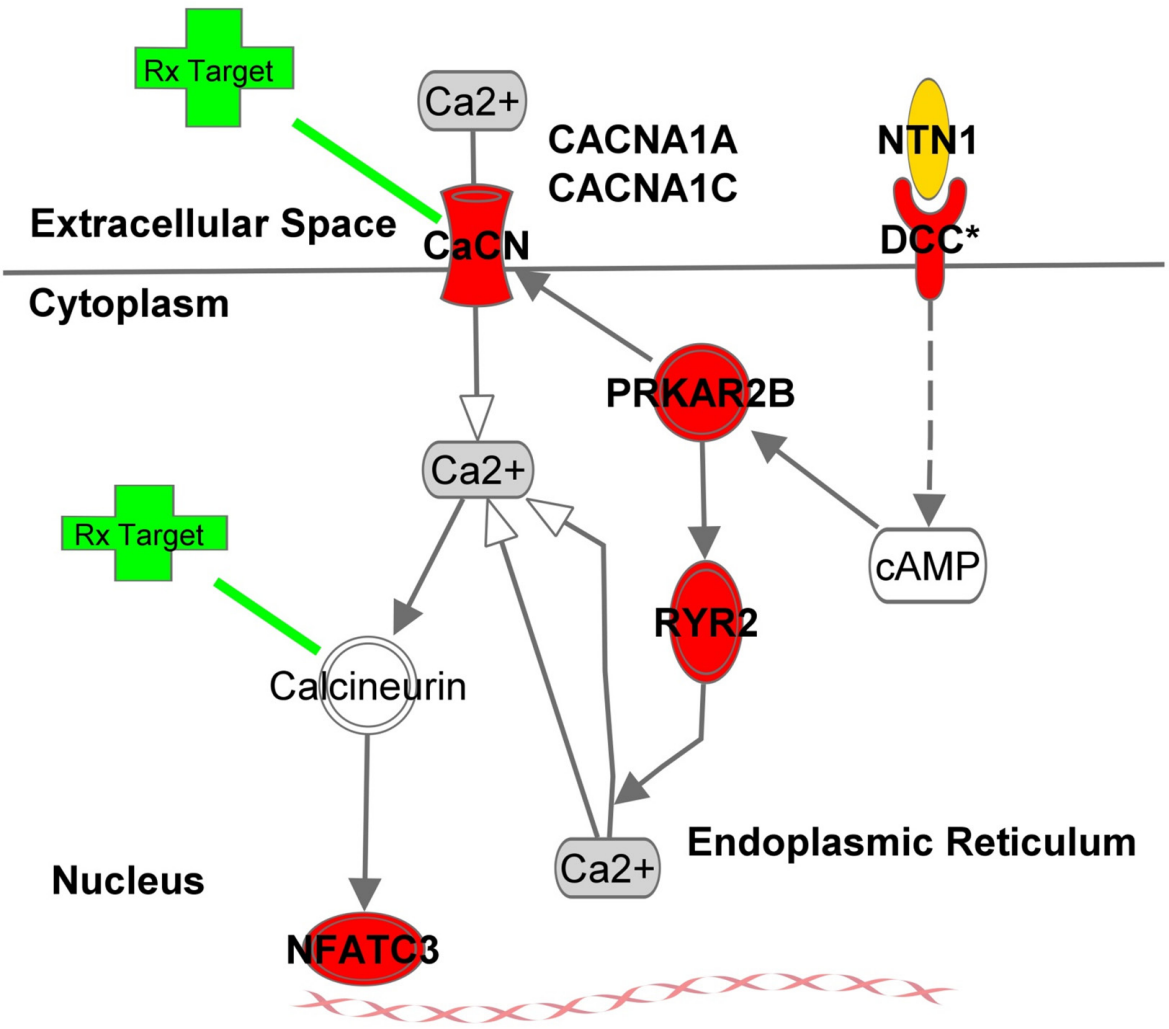
Netrin-1-Based cAMP Signaling Pathway Converging on NFAT. With the exception of calcineurin, all constituents represented in the figure contain DNA sequence variants associated with advanced AMD at *P*-values < 1.0 x 10^–3^. Full gene names exist in the text and at www.ncbi.nlm.nih.gov/gene/. A list of drugs that target CACNA1A, CACNA1C, and calcineurin exists in **S1 Table**. Diagram was generated with INGENUITY Systems products and is based on a curated resource published by INGENUITY Systems and QIAGEN (Netrin Signaling Pathway, IPA Version: 260639, Content Version: 18030641, Release Date: 6 December 2013).

Since netrin-1 demonstrates a capacity to modulate cell survival and rescue in microenvironments similar to those of the fovea, we investigated single-marker and aggregate SNP-based associations of advanced AMD (AAMD) with DNA variants resident within a curated set of genes (see Methods) encoding netrin-1 signaling pathway constituents. Within a 77,000-person cohort, tested as part of an international project on the molecular genetics of AMD, advanced AMD-associated SNPs existed in genes for netrin-1 (*NTN1*), *DCC*, and 6 other netrin-related genes. Pathway-based analysis demonstrated robust enrichment of netrin-1 pathway constituents with AMD-associated SNPs—particularly in a sub-pathway acting, after netrin-1 binding of DCC, through a 3'-5'-cyclic adenosine monophosphate (cAMP)-calcineurin (CN)- nuclear factor of activated T-cells (NFAT) axis (**Fig 1**). FDA-approved drugs targeting calcium channels and a calmodulin-dependent phosphatase in the cAMP arm of the netrin-1-DCC pathway now exist. These results provide a reasonable basis to initiate applied clinical research examining the role of netrin-1-based signaling in AMD for the purpose of developing promising preventive or therapeutic approaches.

## Methods

The National Eye Institute-granted AMD Gene Consortium (AGC) has published data on AMD phenotype and DNA sequence variation covering the human genome (http://nih.gov/news/health/mar2013/nei-03.htm).[12] All research involving human participants at the 18 participating AGC centers was approved by institutional review boards. At each of these centers all clinical investigation was conducted according to the principles expressed in the *Declaration of Helsinki*. Informed consent was obtained from all participants. Details of human subjects procedures, IRB approvals, and references to consenting processes for each of the AGC centers exists at: http://www.nature.com/ng/journal/v45/n4/extref/ng.2578-S1.pdf —pp. 30–41. All data have been de-identified. We tapped the public-access AGC resource [12] for the purpose of testing *a priori* hypotheses on the putative roles of *NTN1*, *NTN1-*related genes, and NTN1-based pathways in AMD.

### Data Sources & Subjects

The analytic sample contained >17,100 advanced AMD cases and >60,000 age- and sex-matched controls of European and Asian ancestry. Controls were AMD-free. All findings passed quality assessment tests for completeness of genotyping and Hardy-Weinberg equilibrium. Details on methods and quality control procedures exist in Fritsche *et al*.[12]

### Extant Findings and the Analytic Approach

In the original application of the AGC data, relationships of AMD with more than 3 million common sequence variants were evaluated using a *hypothesis-free* approach. The false positive rate test criterion (*P*-value), adjusted for multiple analyses on a type I error of 0.05 using the Bonferroni correction was set at ~5.0 x 10^–8^. **S1 Fig** is a plot of 19 loci attaining significance using this highly conservative genome-wide test criterion to reduce the probability of even a single false discovery. Genomic regions most strongly associated with AMD existed in chromosome regions 1q31 (containing a cluster of complement factor H genes) and 10q26 (the HtrA serine peptidase 1 (HTRA1)/age-related macular susceptibility 2 (ARMS2) region), with respective *P*-values of 1 x 10^–283^ and 4 x 10^–353^. Other strong relationships existed for SNPs in loci overlapping complement factor 3 (*C3*), complement factor I (*CFI*), and vascular endothelial growth factor A (*VEGFA*)—these yielded *P*-values of 2 x 10^–26^, 2 x 10^–10^, and 4 x 10^–12^, respectively.

Ward and Kellis discuss evidence demonstrating that common SNPs associated with complex traits at *P*-values far below those significant at a genome-wide level have the capacity to contribute small, but meaningful effects to phenotypic variation.[13] DNA sequence variants in *NTN1* and netrin-related genes showed moderate effect sizes for relationships with AAMD in the AGC results. The aggregate effects of such sequence variants may be notable. **S1 Fig** contains plots of the AAMD-associated NTN1-related genes superimposed on findings from the original genome-wide study.[12] SNPs in *NTN1* and *DCC* showed the strongest relationships within the NTN1 gene set. As neither of these genes are resident in chromosomes housing any of the 19 loci manifesting the AGC genome-wide associations [12], we initiated the current project for the purpose of examining the putative role of a biologically plausible signaling pathway with the capacity to affect the neural and vascular retina.

### Nested Study Design

**Fig 2:**
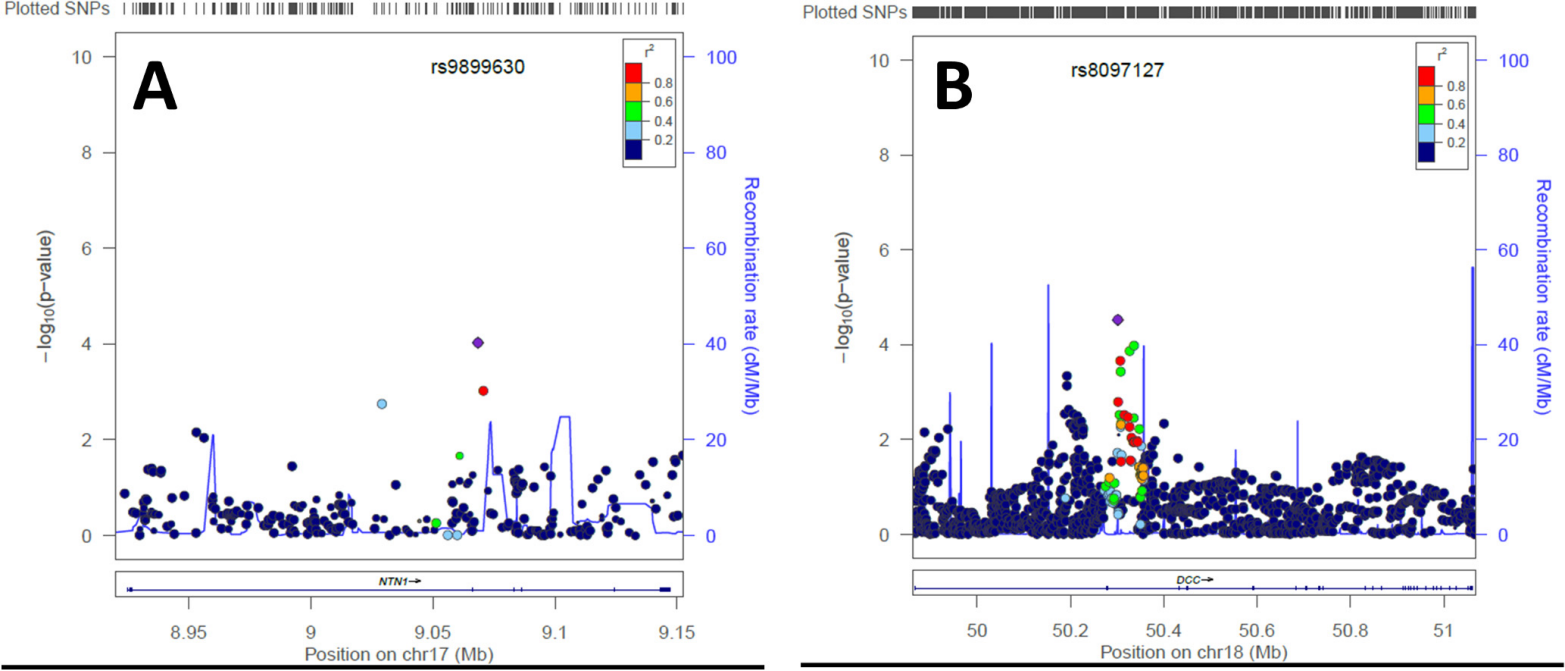
Association findings for advanced age-related macular degeneration-SNP relationships in (A) Netrin-1 (*NTN1*) and (B) Deleted in Colorectal Cancer (*DCC*) genes. Points are colored to represent level of linkage disequilibrium (see r^2^ in legend) with *NTN1* rs9899630 and *DCC* rs8097127 (these two SNPs are represented by violet symbols). SNP genotyping density is represented by the hatch marks at the top of the figure. Positional coordinates of genes and SNPs are labeled on the bottom of the figure. Exons are represented within the gene boundaries with vertical bars. Data are from the AMD Gene Consortium cohort.[12] The figure was generated with LocusZoom[24].

We applied a three-phase design, involving a nested candidate gene study and pathway analysis on *NTN1* results from the AGC whole-genome database. In Phase 1 we determined whether DNA sequence variants in *NTN1* were associated with advanced AMD. *A priori* hypotheses were tested on 255 *NTN1*-resident sequence variants. The Bonferroni corrected *P*-value for multiple testing, was 1.96 x 10^–4^ (α = 0.05/255 ‘independent’ tests). The denominator used to compute this highly conservative value is based on the assumption that none of the tested *NTN1*-resident SNPs are co-inherited; in actuality, many of these SNPs are in nearly complete linkage disequilibrium (**Fig 2**). After confirming existence of an AAMD-*NTN1* relationship at *P* < 9.48 x 10^–5^ (see *Results*) we made the logical progression in Phase 2 to investigate the possible association of AAMD with *DCC*, the gene encoding the primary netrin-1 receptor. We examined 1381 *DCC*-resident SNPs from the AGC data. The Bonferroni-corrected *P*-value criterion for *a priori* hypothesis testing on multiple analyses of *DCC* was 3.62 x 10^–5^. In support and extension of the *NTN1* finding, a significant AMD-*DCC* relationship also existed (*P* < 3.03 x 10^–5^, see *Results*). From this point we computed the false discovery rate (FDR *Q*-value), to estimate the expected proportion of false positives among significant tests for *NTN1* and *DCC*. This effort also yielded significant findings (see *Results*section). The plausibility of inferences from these robust statistical findings, taken in the biologic context of a NTN1-DCC receptor relationship and extant literature on the association of NTN1 with physiologic processes affecting retinal heath, led us to implement the third phase of analyses: testing for enrichment of AMD-associated SNPs within *NTN1*, *DCC*, and other genes encoding constituents of a netrin-1-based signaling pathway.

### Data Analysis

Single markers were tested for association with AAMD using additive models.[12] We evaluated Phase 1 and Phase 2 AAMD-SNP findings with Bonferroni-adjusted *P*-values and *Q*-values (the latter were computed with *QValue* software package, available at genomics.princeton.edu/storeylab/qvalue/). We used INRICH, a pathway analysis method developed by Lee *et al*.[14], to examine linkage disequilibrium (LD)-independent genomic intervals for common AMD-associated DNA sequence variants within a group of 49 genes encoding constituents of netrin-1-based systems. INRICH is a multi-locus method that uses positional clustering of SNPs to account for genomic confounding factors (such as varying gene size, SNP density, LD, and local clustering of functionally related genes). AMD-associated LD-independent genomic intervals containing 1-or-more AMD-related SNP(s) were defined from the positional coordinates of SNPs attaining *P*-values < 0.00149 in the AGC dataset. In *NTN1*, *DCC* and most NTN1-related genes we studied, this *P*-value threshold was associated with FDRs < 0.12. AMD-associated LD-independent genomic intervals were computed using tag SNP selection tools in PLINK (r^2^ = 0.60, associated SNPs < 20kb in distance from the tag SNP). We applied 100,000 permutations in our first phase of netrin-1-based system enrichment analysis and 10,000 in the bootstrap replication phase for correction of multiple testing. Epistasis was tested on data from the NEI Study of Age-Related Macular Degeneration (NEI-AMD, dbGaP Project 000182) with PLINK, as described in SanGiovanni *et al*. [15]

### Gene Set

The netrin-1 gene set was obtained from a curated resource published by INGENUITY Systems and QIAGEN (Netrin Signaling Pathway, IPA Version: 260639, Content Version: 18030641, Release Date: 6 December 2013). **S2 Fig** shows elements of the gene set in the context of this signaling pathway.

## Results

AAMD-associated sequence variants existed in genes encoding *NTN1* (rs9899630 *P* < 9.48 x 10^–5^) and *DCC* (rs8097127 *P* < 3.03 x 10^–5^). Respective *Q*-values for these *NTN1* and *DCC* SNPs are 0.019 and 0.029, indicating that the expected proportion of false positives among significant tests was less than 3-in-100. Similar results existed for neovascular (NV) AMD and geographic atrophy (GA), although *NTN1*- and *DCC*-resident NV AMD-associated SNPs were not co-inherited at high frequency (r^2^ > 0.80) with those of respective GA-associated SNPs. Findings existed for 4486 people with NV AMD and 1925 people with GA. The AAMD endpoint yielded most significant findings in NTN1-related genes.

Pathway analysis with INRICH[14] showed enrichment with AAMD-associated SNPs resident in 9 linkage disequilibrium (LD)-independent loci overlapping *NTN1*, *DCC*, and 6 other genes (corrected *P* = 0.038). Despite statistically significant relationships of *NTN1* and *DCC* SNPs with NV AMD and GA phenotypes, the significance of pathway enrichment findings persisted after correction for multiple testing in the case of the AAMD only. **Table 1**contains details for AAMD-associated SNPs in netrin-1 pathway-associated genes. Genes in this set associated with AAMD at *P*-values < 1.49 x 10^–3^ included: 1) voltage-dependent calcium channels—P/Q type, alpha 1A subunit (*CACNA1A*, rs4926262, *P* < 8.30 x 10^–4^, *Q* < 0.236) and L type alpha 1C subunit (*CACNA1C*, rs3819526, *P* < 2.19 x 10^–4^, *Q* < 0.063) involved in NTN1-based cyclic nucleotide cAMP and cGMP pathways; 2) the ryanodine receptor 2 (RYR2, rs2177065, *P* < 8.54 x 10^–4^, *Q* < 0.717) a component of a tetrameric calcium channel that interacts with CACNA1A and CACNA1C in the NTN1-based cAMP pathway; 3) nuclear factor of activated T-cells, cytoplasmic, calcineurin-dependent 3 (*NFATC3*, rs7192187, *P* < 7.80 x 10^–4^, *Q* < 0.010), the nuclear target of the NTN1-based cAMP pathway containing CACNA1A, CACNA1C, and RYR2; 4) actin binding LIM protein family, member 3 (*ABLIM3*, rs4361503, *P* < 5.29 x 10^–4^, *Q* < 0.057) a member of a NTN1-based Rho-GTPase pathway implicated in actin dynamics and growth cone attraction; and, 5) protein kinase cGMP-dependent type I (*PRKG1*, rs1444402, *P* < 1.72 x 10^–4^, *Q* < 0.112), a gene encoding soluble I-alpha and I-beta forms of a kinase constituent of a nitric oxide-cGMP signaling pathway involved in regulation of neuronal function—cGMP-dependent protein kinases are associated with photoreceptor death in model systems.

**Table 1:** Advanced AMD-associated SNPs in genes encoding Netrin-1 related proteins.

Symbol	Alleles	SNP	*P*-value	*Q*-value	Protein Function
NTN1	A|G	rs9899630	9.48 x 10^–5^	0.019	Netrin-1
DCC	A|G	rs8097127	3.03 x 10^–5^	0.029	Netrin-1 Receptor
PRKG1	A|G	rs1444402	1.72 x 10^–4^	0.112	cGMP Signaling
CACNA1C	T|C	rs3819526	2.19 x 10^–4^	0.063	cAMP Signaling
CACNA1A	A|G	rs4926262	8.30 x 10^–4^	0.236	cAMP Signaling
ITPR2	T|C	rs3816834	6.20 x 10^–4^	0.119	cAMP Signaling
NFATC3	T|C	rs7192187	7.80 x 10^–4^	0.010	cAMP Signaling
RYR2	A|G	rs2177065	8.54 x 10^–4^	0.717	cAMP Signaling
ABLIM3	T|C	rs4361503	5.29 x 10^–4^	0.057	Rho GTPAse

Full gene names exist at www.ncbi.nlm.nih.gov/gene/. Alleles are listed as risk-associated allele|non-risk-associated allele. *P*-values were computed from additive models (modeling effects of 2|1|0 risk alleles).

In addition to the AAMD-NTN1-DCC-cAMP relationships reported at *P* < 1.49 x 10^–3^, two others existed at *P* < 2.0 x 10^–3^; these were for the netrin-1 receptor Down syndrome cell adhesion molecule (*DSCAM*, rs7279213, *P* < 1.53 x 10^–3^) and protein kinase cAMP-dependent regulatory type II beta (*PRKAR2B*, rs3779500, *P* < 2.10 x 10^–3^).

## Discussion

Our findings support the idea that processes driven by NTN1-DCC binding operate in advanced AMD. These results are aligned with others that demonstrate channeling of NTN-associated molecules [6] for local maintenance of trophic signals in neurovascular crosstalk and a signaling system involved in retinal cell survival and rescue.[8] NTN1-DCC signaling pathways influence cAMP-, cGMP-, and Rho-GTPase-based signaling. We observed AAMD-associated SNPs and an aggregate effect in numerous genes that encode interacting constituents of the NTN1-DCC-cAMP-CN-NFAT axis (**Fig 1**).

In the *Methods* section we presented background on AMD-associated loci emerging from the hypothesis-free whole-genome tests. Common SNPs associated with complex traits at *P*-values far below those significant on a genome-wide level have been demonstrated to contribute small, but meaningful effects in disease risk (reviewed in [13]). Such loci have clustered in genes encoding constituents of biological pathways driving physiologic processes known to be dysregulated in disease.[15, 16] The central premise here is that there is value in examining biologically plausible contributions from sequence variants manifesting moderate-to-weak, but real, effects. The combined influence of such effects may alter risk in complex disease. [14, 17] Our primary approach was to apply a pathway-based enrichment analysis for the purpose of making inferences on the existence of biologically plausible aggregate effects from the NTN1 system.

We have also found examination of epistatic (SNP-SNP) interactions to be informative—both in support of our inferences on pathway relevance and as a link of the netrin-1 system to the most strongly AMD-associated loci in 1q31 and 10q26. We tested the existence of epistatic relationships within the NTN1-DCC-cAMP-CN-NFAT sub-pathway using data from the NEI Study of Age-Related Macular Degeneration (NEI-AMD, dbGaP Project 000182) to find strong NTN1-RYR2 (rs9674710-rs12046077, *P* < 9.88 x 10^–5^; rs4791779-rs4659491, *P* < 1.37 x 10^–5^) and DCC-CACNA1C (rs3915401-rs1009281, *P* < 2.28 x 10^–5^) associations. Others have shown combined effects of sequence variants in *DCC* with loci in 1q31 and 10q26 highlighted by the AGC—in the period during which the present report was under revision Riveros *et al*. published results from an exhaustive genome-wide interaction analysis involving 137,467,878 tests for AMD-associated SNPs.[10] From this work a *DCC*-resident SNP (rs9966348) emerged in a set of 19 highly interconnected DNA sequence variants manifesting significant interactions of the highest magnitude. *DCC* rs9966348 (*P*_*interaction*_ < 7.74 x 10^–13^) showed strong relationships with 12 of the 18 other most strongly associated loci—among these were 3 SNPs in 1q31 and 2 in 10q26. Such findings establish a link between NTN1-based systems and consistently replicated complement system risk loci for AMD. While statistically significant *NTN1*-resident SNPs did not attain *P*-values at levels distinguishing DCC rs9966348 and the other most strongly associated 18 loci, strong interactions existed between NTN1 and SNPs in the 1q31 (*CFHR4*) locus. Riveros *et al*. present the key message that biologically relevant inferences on complex disease should include assessment of putative interactions. The case for *DCC* is pertinent to this point as the combined (interaction) effect of rs9966348 exceeds, by several orders of magnitude, those expected from its individual main effect.

Putative Mechanisms. Products of genes in the NTN1-DCC-cAMP-CN-NFAT sub-pathway are localized to the cell membrane, cytoplasm, endoplasmic reticulum, and nucleus. NFATs are cytosolic phosphoproteins acting, after calcium-induced translocation, as constituents of DNA-binding transcription complexes; NFAT factors were first discovered in T-cells. We observed an AMD-associated SNP in *NFATC3*. NFATC3 is dephosphorylated by calcineurin in response to intracellular calcium—an increased calcium status permits fluent transmission of nuclear signals to which NFATC3 may localize; the result is an accumulation of NFATC3 in the nucleus. NFATC3 exits the nucleus in a tightly controlled manner. In a model system of arterial smooth muscle, nuclear export of NFATC3 was regulated by mitogen-activated protein kinase 9 (MAPK9, a.k.a. JNK2) in an exportin 1-dependent process.[18] Exportin-1 acts as a receptor in the nuclear envelope for leucine-rich nuclear export signal (NES)-dependent protein transport, controlling localization of cyclin B, mitogen-activated protein kinase (MAPK), MAPKAP kinase 2 (MAPKAPK2), NFAT, and jun proto-oncogene (JUN). These findings may have relevance to pathophysiologic processes in the retina, as we have recently reported relationships of AMD with aggregates of sequence variants resident in genes encoding constituents of the JNK/MAPK signaling system, including a sequence variant in the gene encoding MAPKAPK2.[15] NFATC3 influences mesenteric arterial remodeling in response to systemic intermittent hypoxia in mice[19] and has been shown as a prostaglandin-endoperoxide synthase 2 (a.k.a. cyclooxygenase 2 or COX2) transcription regulator and requisite factor for COX2-dependent migration and angiogenesis in vivo knock-down murine models.[20] Relevance for the present work is that transcriptional inhibition of COX2 with a CN inhibitor (*viz*. affecting the phosphatase directly upstream of NFAT3) blocks VEGF-dependent angiogenesis in vivo.[21] NFATC family members have been tested in models of pathogenic retinal angiogenesis by Bretz *et al*.[22] for their influence on retinal neovascularization within a model of oxygen-induced retinopathy. The focus of work in this report was on NFATC1 because, in human retinal microvascular endothelial cells, this isoform of NFAT clearly translocated to the nucleus after 30 minutes of exposure to vascular endothelial growth factor (n.b. all four calcineurin-dependent NFAT isoforms displayed immunoreactivity in a translocalization test). In the subsequent *in vivo* experiments NFATc1 regulated VEGF expression and acted as a downstream target of VEGF signals in the retina—inhibition of NFAT, *via* blockade of serine phosphatase calcineurin with the small organic molecule inhibitor of NFAT-calcineurin association-6 (INCA-6) and tacrolimus (FK-506), reduced the severity of oxygen-induced retinal neovascularization in model systems by 70%. Tacrolimus complexes with immunophilin FKB12, a molecule that binds CN to inhibit CN activation and associated phosphatase activity—through this process nuclear translocation of NFAT is then inhibited. Bretz *et al*. note that NFAT transcription factor binding sites exist on genes encoding numerous proteins implicated in pathologic retinal angiogenesis; these include hypoxia inducible factor alpha (*HIF1A*)—a sensor of cellular and physiologic stresses leading to retinal neovascularization, VEGF receptor 1 (fms-related tyrosine kinase 1, *FLT1*) —a target of vascular permeability factor, tissue factor (*F3*)—a trigger for angiogenic and inflammatory responses[23], and matrix metalloproteinases 2 and 9 (*MMP2*, *MMP9*)—molecules acting in angiogenesis-related ECM remodeling. A list of drugs targeting constituents of the NTN1-DCC-cAMP-CN-NFAT axis genes exists in **S1 Table**.

The putative role of NTN1-DCC binding in AMD is most intriguing to us because the demonstrated capacity of these molecules exists for a highly localized influence on cell migration, cell-substrate adhesion, elaboration of vascular networks, inflammation, and protein translation[7]. These processes may be triggered by altered metabolism and inflammation in retinal neurons, glia, immune cells and vessels affected in AMD and may explain the adaptive capacity for a sparing pattern of the fovea until late stages of the disease. The influence of netrin-driven responses on *localized* hypoxia-induced inflammation has been examined with *in vivo* models in epithelium of mucosal organs to demonstrate the role of netrin in regulation of inﬂammatory cell migration.[11] If these findings can be linked to the channeling of retinal resources[8] and the efficacy of tacrolimus and INCA-6 in animal models of retinal neovascularization[22], additional study of netrin-based signaling in the retina will likely elucidate promising venues for applied clinical research on AMD prevention and treatments.

## Supporting Information

S1 Fig Chromosomal ideogram plotting advanced AMD (AAMD)-associated loci published by Fritsche *et al*.[12] (blue symbols) and AAMD-associated Netrin-1 (*NTN1*) related loci (green symbols). *NTN1*-related loci are as follows: Chromosome 1, *RYR2*; Chromosome 5, *ABLIM3*; Chromosome 10, *PRKG1*; Chromosome 12, *CACNA1C*; Chromosome 16, *NFATC3*; Chromosome 17, *NTN1*; Chromosome 18, *DCC*; Chromosome 19, *CACNA1A*. Full gene names exist at www.ncbi.nlm.nih.gov/gene/. This figure was generated with PhenoGram (ritchielab.psu.edu/software/).
(PNG)

S2 Fig Netrin-1-Based Signaling Pathway. All genes represented by red symbols contain DNA sequence variants associated with advanced AMD at *P*-values < 1.0 x 10^–3^. Full gene names exist in the text and at www.ncbi.nlm.nih.gov/gene/. Diagram was generated with INGENUITY Systems products and is based on a curated resource published by INGENUITY Systems and QIAGEN (Netrin Signaling Pathway, IPA Version: 260639, Content Version: 18030641, Release Date: 6 December 2013).
(JPG)

S1 Table Annotations for AMD-associated sequence variants in the Netrin-1-Based Signaling Pathway. (XLSX)
