# Supplementary figures and images for: Netrin-1 – DCC Signaling Systems and Age-Related Macular Degeneration
Source: PLoS One. 2015 May 7;10(5):e0125548. doi: 10.1371/journal.pone.0125548 (PMC4423995; doi:10.1371/journal.pone.0125548)

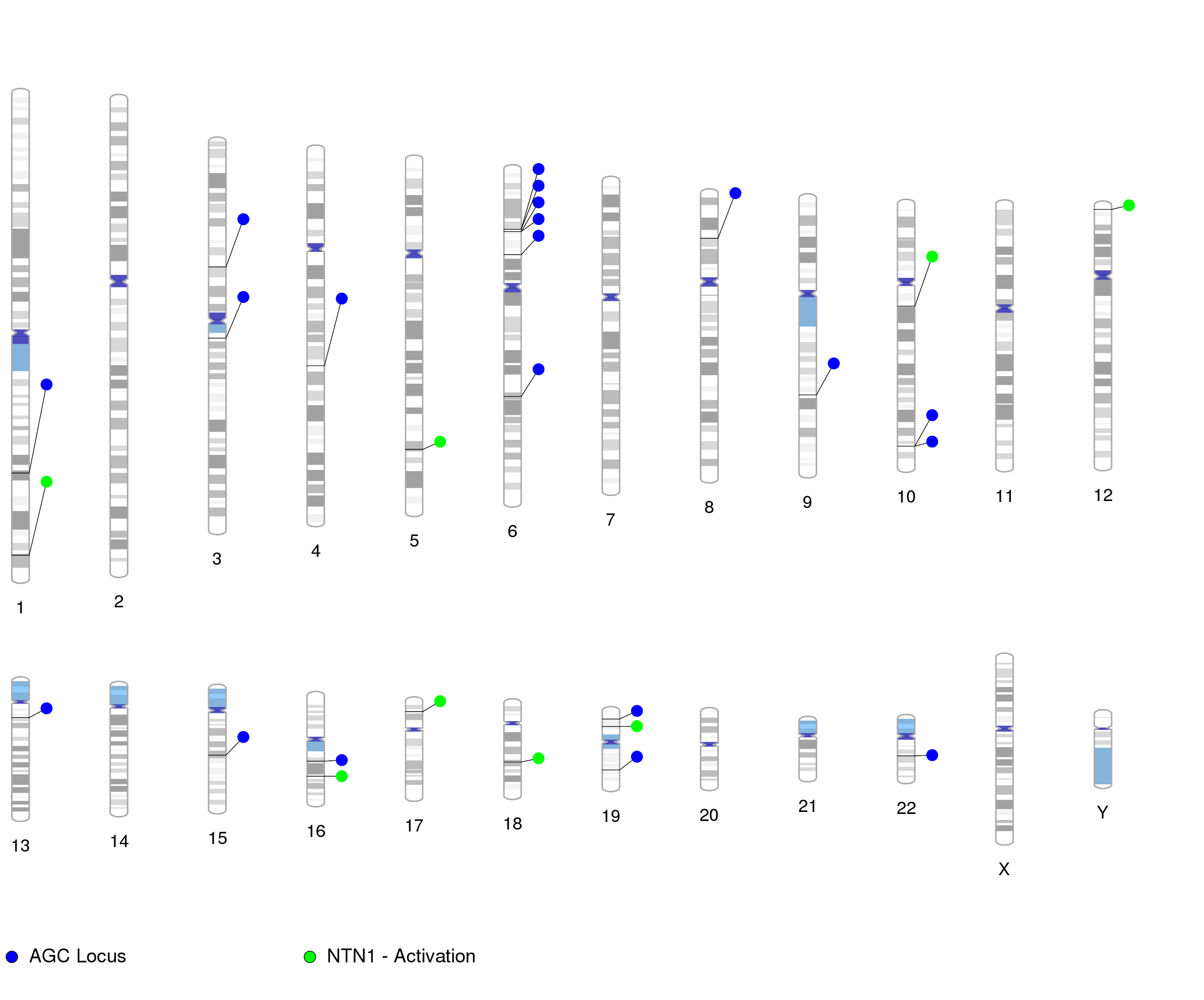

Supplement: S1 Fig — NTN1-related loci are as follows: Chromosome 1, RYR2; Chromosome 5, ABLIM3; Chromosome 10, PRKG1; Chromosome 12, CACNA1C; Chromosome 16, NFATC3; Chromosome 17, NTN1; Chromosome 18, DCC; Chromosome 19, CACNA1A. Full gene names exist at www.ncbi.nlm.nih.gov/gene/. This figure was generated with PhenoGram (ritchielab.psu.edu/software/). (PNG) [file pone.0125548.s001.png]

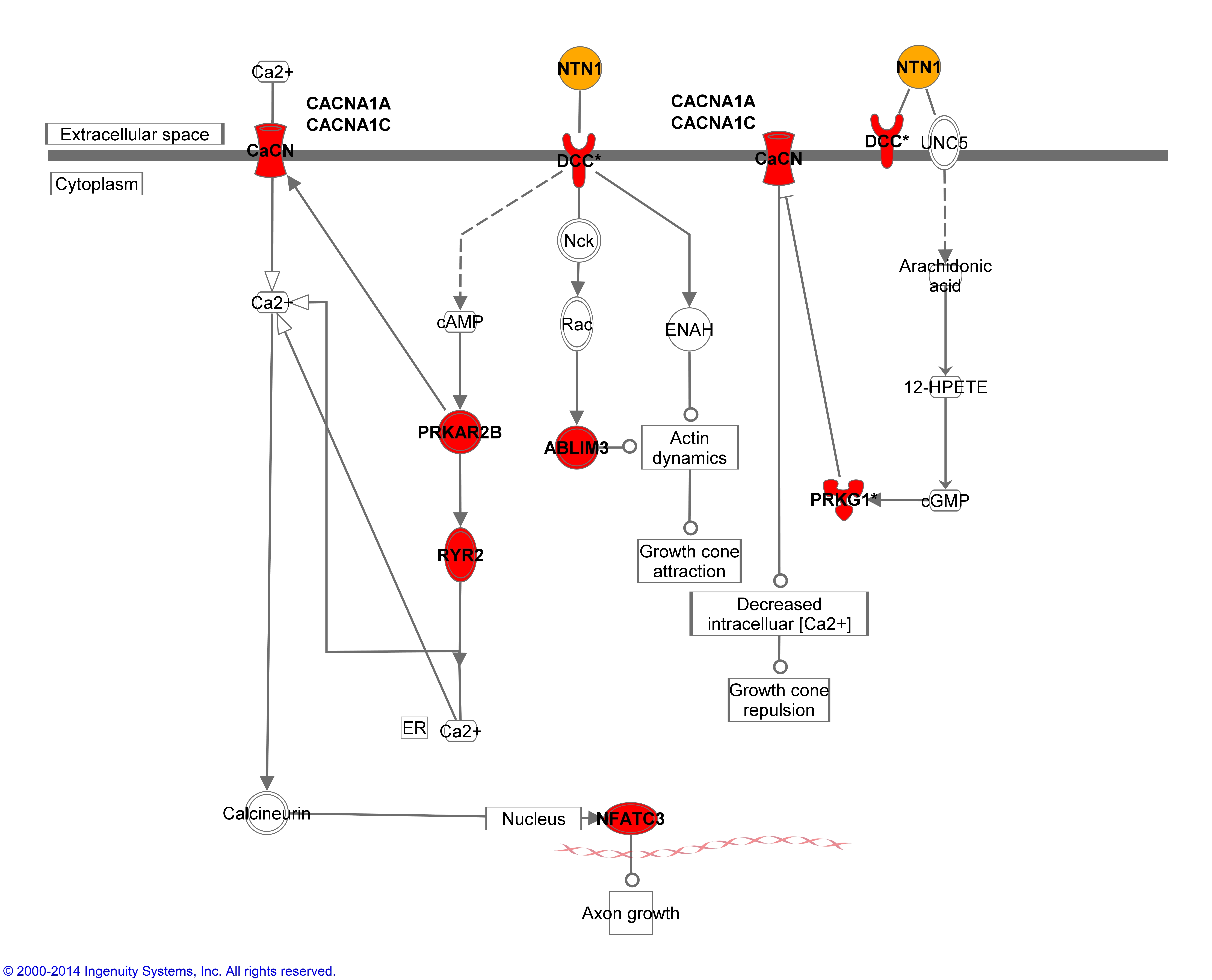

Supplement: S2 Fig — All genes represented by red symbols contain DNA sequence variants associated with advanced AMD at P-values < 1.0 x 10^–3^. Full gene names exist in the text and at www.ncbi.nlm.nih.gov/gene/. Diagram was generated with INGENUITY Systems products and is based on a curated resource published by INGENUITY Systems and QIAGEN (Netrin Signaling Pathway, IPA Version: 260639, Content Version: 18030641, Release Date: 6 December 2013). (JPG) [file pone.0125548.s002.jpg]
